# Development of an adipocyte differentiation protocol using 3T3-L1 cells for the investigation of the browning process: identification of the PPAR-γ agonist rosiglitazone as a browning reference drug

**DOI:** 10.3389/fphar.2025.1546456

**Published:** 2025-04-14

**Authors:** Lorenzo Flori, Giulia Galgani, Giorgia Bray, Chiara Ippolito, Cristina Segnani, Carolina Pellegrini, Valentina Citi, Nunzia Bernardini, Alma Martelli, Vincenzo Calderone

**Affiliations:** ^1^ Department of Pharmacy, University of Pisa, Pisa, Italy; ^2^ Department of Clinical and Experimental Medicine, Unit of Histology and Medical Embryology, University of Pisa, Pisa, Italy; ^3^ Interdepartmental Research Centre of Ageing, Biology and Pathology, University of Pisa, Pisa, Italy; ^4^ Center for Instrument Sharing of the University of Pisa (CISUP), University of Pisa, Pisa, Italy

**Keywords:** browning, beige adipocyte, white adipocyte, adipocyte differentiation, 3T3-L1, PPAR-γ, rosiglitazone, isoproterenol

## Abstract

**Background:**

Obesity is a metabolic disease that is characterized by an excessive accumulation of adipose tissue (AT) and is often associated with other pathologies. AT is a lipid storage organ with endocrine functions that presents two main phenotypes: white adipose tissue (WAT) and brown adipose tissue (BAT). Preadipocytes or mature white adipocyte cells can differentiate in a middle phenotype with morpho/functional characteristics between WAT and BAT, known as brown-like or beige adipose tissue (BeAT), through the browning process. Considering the interest in stimulating the browning process in metabolic disorders and the lack of clarity, evenness, and reproducibility of the preclinical models, the detailed description of an adipocyte differentiation protocol and the “*de novo*” development of a beige adipocyte phenotype has been described. Furthermore, the most described stimuli in inducing the browning process, such as PPAR-γ agonists (using rosiglitazone, RGZ) and β-adrenergic stimulators (using isoproterenol, ISO), were evaluated in order to describe their involvement in the browning process and identify a reference compound for the induction of the “*de novo*” browning.

**Methods:**

Immortalized murine embryonic fibroblasts (3T3-L1) cells were differentiated for up to 17 days using a differentiation medium (DM) and a maintenance medium (MM) with or without RGZ or ISO to obtain both the mature white and the beige adipocyte phenotype. The differentiation was evaluated by the Oil Red O (ORO) staining assay, citrate synthase activity, and mitochondrial uncoupling protein 1 (UCP-1) immunodetection and expression performed on different days (T0, T3, T10, and T17) after the induction of differentiation.

**Results:**

The results indicated that RGZ induced morphology and ORO-positive lipid deposits and increased the activity of citrate synthase enzyme and UCP-1 levels overlapping with a beige adipocyte phenotype after 17 days. ISO did not display a significant effect in these experimental conditions.

**Conclusion:**

Overall, this work describes in depth the different phases of the adipocyte differentiation process by offering a detailed and reproducible “*de novo*” browning differentiation model. Furthermore, the efficacy of the stimulation of the PPAR-γ pathway in obtaining a beige adipocyte phenotype demonstrates that RGZ can induce the browning process and elects it as a perfect reference compound for experimental procedures in this field.

## 1 Introduction

Metabolic disorders are complex and multifactorial pathologies with morphological and functional alterations affecting different organs and tissues. In this context, obesity is a chronic metabolic disease increasing globally, identified by a body mass index (BMI) value greater than 30 kg/m^2^ and closely related to the increase of body fat (Roberto et al., 2015; Yang et al., 2022; Kawai et al., 2021). Obesity is characterized by qualitative and quantitative alterations of AT, leading to the development of comorbidities, such as type 2 diabetes, dyslipidemia, cardiovascular alterations, hypertension, and some types of cancer (Després and Lemieux, 2006; Monteiro and Azevedo, 2010; Madan et al., 2023).

In this context, the traditional definition of AT as a passive deposit of energy substrate has long been outdated. The production of hormones, such as adipsin and leptin, discovered in the early 90s, has definitively established its investiture as an endocrine organ (Mohamed-Ali et al., 1998; Kershaw and Flier, 2004; Prins, 2002). AT expresses various target receptors that mediate afferent signals, resulting in the secretion of bioactive peptides (adipokines and cytokines) with endocrine (systemic) or autocrine/paracrine (local) activities. AT influences many physiological processes such as glucose uptake, lipid metabolism, blood pressure homeostasis, inflammation, and immune responses (Šimják et al., 2020; Stanford et al., 2012; Ferrannini et al., 2018; Scheja and Heeren, 2019; Unamuno et al., 2018; Xu et al., 2024; Ghigliotti et al., 2014; Stolarczyk, 2017).

The two main phenotypes of AT are characterized by variability in origin, morphology, localization, and function (Zwick et al., 2018; Bartelt and Heeren, 2014; Hilton et al., 2015). WAT is a dynamic organ engaged in the storage and consumption of lipids and endocrine signaling. Generally, it is located in the visceral zone (vWAT) and the subcutaneous area (sWAT). WAT is mainly composed of undifferentiated preadipocyte cells and mature white adipocytes. White adipocytes are characterized by large lipid droplets covering most of the cell volume while possessing few mitochondria (Sabaratnam et al., 2023).

In contrast, BAT is located in the supraclavicular, paravertebral, and periadrenal regions and contributes to regulating energy homeostasis. It is characterized by adipocytes rich in mitochondria and containing multiple small lipid droplets (Cannon and Nedergaard, 2004). Instead of storing and releasing energy, BAT uses substrates for non-shivering thermogenesis (Silva, 2011). The thermogenic activity is mediated by peroxisome proliferator-activated receptor-γ coactivator 1α (PGC-1α), which upregulates mitochondrial biogenesis and the mitochondrial uncoupling protein 1 (UCP-1) expression, which decouples respiration from the production of ATP “switching the toggle” on heat production (Chouchani and Kajimura, 2019; Brownstein et al., 2022).

WAT depots also “host” a third adipocyte phenotype with morpho/functional characteristics between WAT and BAT, known as brown-like or BeAT (Pilkington et al., 2021). Beige adipocytes derive from adipocyte progenitor cells (APCs) that do not express myogenic factor 5 (Myf-5) or PR domain containing 16 (PRDM16) (both existing in WAT and migrating from other tissues) through a process called “*de novo*” differentiation, or from the transdifferentiation of mature white adipocytes (Seale et al., 2009; Granneman et al., 2005; Himms-Hagen et al., 2000). Both these differentiation processes are called browning.

UCP-1 is considered the central marker in the phenotypic discrimination between thermogenically active ATs such as BAT and BeAT and non-thermogenically active tissues such as WAT. UCP-1 is a protein localized in the mitochondrial inner membrane and generates heat by dissipating the energetic proton gradient from the electron transport chain in mitochondrial respiration (Ikeda and Yamada, 2020). In physiological conditions, the balance between WAT and BeAT depots is guaranteed and dynamically regulated according to the energy needs of the organism. However, in patients with obesity, the metabolic dynamism of AT is strongly influenced by hypertrophic and hyperplastic processes, altering not only the lipid storage and energy production but also influencing the metabolic homeostasis and functionality of distal organs. The browning process is also negatively influenced by the chronic, low-grade inflammatory state, called metaflammation, associated with fat overload (Villarroya et al., 2018). The accumulation of lipids leads to mitochondrial dysfunction, overexpression of pro-inflammatory cytokines, such as tumor necrosis factor α (TNF-α) or interleukin 1β (IL-1β), inhibition of proliferator-activated receptor γ (PPAR-γ), decrease of UCP-1 expression, and macrophage infiltration of M1 pro-inflammatory phenotype (Hotamisligil, 2017; Sakamoto et al., 2016; Roberts‐Toler et al., 2015; Nøhr et al., 2017). Moreover, BAT-deficient mice show diabetic and obese phenotypes at room temperature (Lowell et al., 1993), confirming the importance of thermogenically active adipocytes. Interestingly, recent experimental evidence describes a UCP-1-independent thermogenic pathway that may intervene as a compensatory mechanism (Keipert et al., 2015; Ukropec et al., 2006).

These findings, therefore, confirm that, although there are thermogenic adaptation mechanisms independent of UCP-1, the presence of thermogenically active adipocyte phenotypes such as the beige one are essential in metabolic regulation, confirming the importance of the browning process in physiological/pathological conditions (Ikeda and Yamada, 2020; Golozoubova et al., 2006).

The browning process can be triggered by different stimuli such as physical exercise, exposure to low temperatures (Mu et al., 2021; Lim et al., 2012), and, as hypothesized and partly demonstrated more recently, also through specific molecular targets involved in the adipogenesis process such as PPAR-γ, triiodothyronine (T3), AMP-activated protein kinase (AMPK), sirtuin 1 (SIRT-1), and β-adrenergic stimulation (Zhang et al., 2023; Huang et al., 2024; Lee et al., 2023a; Flori et al., 2023; Machado et al., 2022). In this context, the pharmacological modulation of the browning process attracts an ever-increasing interest in metabolic diseases such as obesity and more complex metabolic syndromes associated with several comorbidities.

Preclinical experimental models, and in particular cellular models, play a key role in the early investigational phases and in screening processes to evaluate the efficacy of molecules of different natures and origins to activate the browning process. To date, immortalized murine embryonic fibroblasts (3T3-L1) are one of the most used cellular models for evaluating the modulation of adipogenesis processes and the molecular mechanisms involved. 3T3-L1 cells represent an excellent model of preadipocyte cells for reproducing adipocyte differentiation protocols (Zebisch et al., 2012; Cave and Crowther, 2019; Takanezawa et al., 2023; Zhang et al., 2022; Chen et al., 2020).

Several experimental protocols of adipocyte differentiation differ in timing, cocktails of differentiation and maintenance media, and reproducibility of the different steps and of the adipocyte phenotype obtained. Furthermore, almost all the protocols used are aimed at evaluating the modulation of the adipogenesis process without closely investigating the browning process and without defining any reference compound capable of being utilized as a positive control for the induction of the browning process and the achievement of a beige adipocyte phenotype (Lee et al., 2023a; Lee et al., 2023b; Kim et al., 2020; Choi et al., 2021; Seo et al., 2018; Molinari et al., 2021). To this end, therefore, this experimental work aims to define the aspects listed above in order to establish an adipocyte differentiation protocol using 3T3-L1 cells for the investigation of the modulation of the *de novo* browning process and the molecular targets involved. Furthermore, this work also aims to provide sufficient evidence to identify a reference compound to be used as a positive control in the induction of the *de novo* browning process and to obtain a mature beige adipocyte phenotype by searching among PPAR-γ agonists (using rosiglitazone) and β-adrenergic stimulators (using isoproterenol), identified by recent experimental evidence as two of the most involved targets in the stimulation of the browning process (Asano et al., 2014; Miller et al., 2015).

## 2 Materials and methods

### 2.1 Differentiation of 3T3-L1 preadipocytes into mature adipocytes

3T3-L1 cells (ATCC, United States) were cultured in basal medium (BM), composed of Dulbecco’s Modified Eagle’s Medium-high glucose (DMEM-HG, Merck KGaA, Germany) supplemented with 10% bovine calf serum (BCS, ATCC, United States) and 1% penicillin-streptomycin with 10,000 units penicillin and 10 mg streptomycin/mL (P/S, Merck KGaA, Germany), in a humidified incubator at 37°C with 5%–10% CO_2_. When the confluence was reached, 15.000 cells/well were seeded in two different supports (24 multiwell plates or 8-well glass chamber slide). The cells were maintained until an over-confluent stage (2 days after reaching confluence) at 37°C, 90% humidity, and 5% CO_2_ to initiate growth arrest and ensure the optimal transition from pre-adipose to adipose-like phenotype (Zebisch et al., 2012; Pacifici et al., 2020; Recinella et al., 2023). Then, BM was removed and replaced with an appropriate volume of 3T3-L1 DM (Tables 1, 2) and incubated for 3 days (T0 of the differentiation procedure). At T3, DM was replaced with MM (Tables 1, 2) and incubated for 2 days. The cells were fed every 2–3 days using 3T3-L1 MM until they were ready for assay at the selected time points of 10 days and 17 days (T10 and T17).

**TABLE 1:** Feeding volumes used at different times for different support. RGZ = rosiglitazone; ISO = isoproterenol; BM = basal medium; DM = differentiation medium; MM = maintenance medium.

Feeding volumes									
24 multiwell plate									
	T −4	T0	T3	T5	T7	T10	T12	T14	T17
Remove (µL)		600 µL	600 µL	800 µL	800 µL	800 µL	800 µL	800 µL	800 µL
Add (µL)	600 µL BM (seeding)	1,000 µL DM	800 µL MM	800 µL MM	800 µL MM	800 µL MM	800 µL MM	800 µL MM	800 µL MM
8-well glass chamber slide									
Remove (µL)		300 µL	180 µL	240 µL	240 µL	240 µL	240 µL	240 µL	240 µL
Add (µL)	300 µL BM (seeding)	300 µL DM	240 µL MM	240 µL MM	240 µL MM	240 µL MM	240 µL MM	240 µL MM	240 µL MM

**TABLE 2:** Culture media composition. RGZ = rosiglitazone; ISO = isoproterenol; BM = basal medium; DM = differentiation medium; MM = maintenance medium; DMEM-HG = Dulbecco’s modified Eagle’s medium-high Glucose; BCS = bovine calf serum; P/S = penicillin-streptomycin; IBMX = 3-isobutyl-1-methylxanthine; DEX = dexamethasone; DMSO = dimethyl sulfoxide.

Culture MEDIA					
	White	RGZ 0.1 µM	RGZ 1 µM	ISO 0.1 µM	ISO 1 µM
**BM**	DMEM-HG + 10% BCS +1% P/S				
**DM**	BM + Insulin (10 μg/mL) + IBMX (0.5 mM) + DEX (2.5 µM) + Vehicle (DMSO)	BM + Insulin (10 μg/mL) + IBMX (0.5 mM) + DEX (2.5 µM) + RGZ 0.1 µM	BM + Insulin (10 μg/mL) + IBMX (0.5 mM) + DEX (2.5 µM) + RGZ 1 µM	BM + Insulin (10 μg/mL) + IBMX (0.5 mM) + DEX (2.5 µM) + ISO 0.1 µM	BM + Insulin (10 μg/mL) + IBMX (0.5 mM) + DEX (2.5 µM) + ISO 1 µM
	Total amount of DMSO: 1.45%				
**MM**	BM + Insulin (10 μg/mL) + Vehicle (DMSO 1% v/v)	BM + Insulin (10 μg/mL) + RGZ 0.1 µM	BM + Insulin (10 μg/mL) + RGZ 1 µM	BM + Insulin (10 μg/mL) + ISO 0.1 µM	BM + Insulin (10 μg/mL) + ISO 1 µM
	Total amount of DMSO: 1.00%				

**FIGURE 1:**
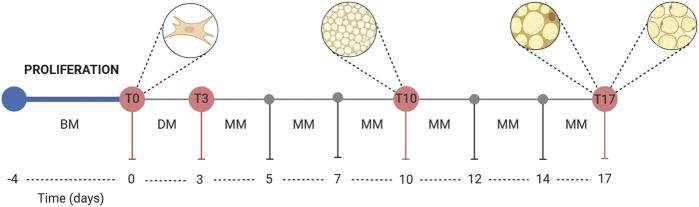
Graphical representation of the adipocyte differentiation process. Abbreviations: BM, basal medium; DM, differentiation medium; MM, maintenance medium.

The DM for obtaining the white adipocyte phenotype contained BM, insulin (10 μg/mL) (Merck KGaA, Germany), three-isobutyl-1-methylxanthine (IBMX, 0.5 mM) (Merck KGaA, Germany), and dexamethasone DEX (2.5 µM) (Merck KGaA, Germany). The MM containing only BM and insulin (10 μg/mL). RGZ (Merck KGaA, Germany) and ISO (Merck KGaA, Germany) at concentrations of 0.1 µM and 1 µM was added to both DM and MM to evaluate their impact on the *de novo* browning process. More details on composition, volumes, and concentrations are reported in Tables 1, 2. A graphical representation of the differentiation process is shown in Figure 1.

### 2.2 Analysis of cellular phenotype and lipid deposits by ORO staining and UCP-1 confocal immunofluorescence

To prepare the staining solution, Oil Red O (ORO), freshly prepared according to Puri et al. (2008), was dissolved in 99% isopropanol (Sigma-Aldrich, 0.3%–0.6% w/v), and three parts of this solution were mixed with two parts of distilled water. Adipocytes were rinsed with phosphate buffer saline (PBS, Sigma-Aldrich) and fixed with 4% formalin. After 1 h of incubation at room temperature, cells were washed first with PBS and then with 60% isopropanol. The lipid depots were dyed by incubation for 20 min with the staining solution. Finally, lipids were observed using an inverted optical microscope (AE 2000, Motic, Hong Kong) equipped with the digital camera VisiCam 3.0 (VWR, United States).

To improve the identification of the 3T3-L1 cell phenotype, we employed immunofluorescence staining for UCP-1 in combination with ORO staining, as reported by Molinari et al. (2021). Briefly, cells were incubated in 0.2% Triton X-100 in PBS, 1% BSA to block nonspecific antibody binding, in rabbit pAb to UCP-1 primary antibody ([1:200] diluted in 1% BSA; AMab10983, Abcam) overnight at 4°C, and then in donkey anti-rabbit Alexa Fluor 488 Plus secondary antibodies (A32790; Molecular Probes, Eugene, OR, United States) for 1 h at RT. After the incubation with ORO and DAPI nuclear counterstaining (62,248, Invitrogen, Life Technologies, Eugene, United States), the slides were mounted using ProLong Diamond (P36961, Invitrogen, Life Technologies, Eugene, United States). Cells were examined by a Leica TCS SP8 confocal laser-scanning microscope (Leica Microsystems, Mannheim, Germany, 40x oil lens) to detect ORO-positive lipid droplets and UCP-1 staining as red and green fluorescent areas, respectively. A sequential confocal scan procedure was used to make representative images of all microscopic fields, and zeta stack acquisition mode with optical sectioning every 0.35 µm was chosen to scan all planes of cells in the z-axis.

The degree of lipid accumulation was evaluated by detecting the ORO-positive lipid droplets by staining, with red pixel areas indicating fat vacuoles, and then dividing the area of droplets by the total area scanned. Staining of all the multiwell/chamber slides was quantified by ImageJ software (Mehlem, Hagberg, Muhl, Eriksson and Falkevall, 2013), and results were expressed as fold change against control (preadipocytes stage). To better understand whether the changes in lipid droplet accumulation depend on the amount of lipid deposit, on the size of the lipid droplets, or both, image analysis was performed to calculate the area of each ORO-positive droplet and their distribution in terms of small, medium, and large lipid droplets (0–120 μm^2^, 120 μm^2^–240 µm^2^, >240 μm^2^, respectively), as reported by Kaczmarek et al. (STAR Protocols 5, 102,977 21 June 2024 ^a^ 2024) (Kaczmarek et al., 2024).

### 2.3 Evaluation of citrate synthase activity and mitochondrial UCP-1 levels

Cells were lysed with a 1% Triton X-100 in PBS (Merck KGaA, Germany) solution. The suspension was centrifuged using a Speed Master 14 R centrifuge (Euroclone, Italy) at 12,000×g for 15 min at 4°C, and the supernatant was recovered.

*Citrate synthase assay*: the Bradford assay was performed to spectrophotometrically measure the protein concentration in the supernatants (EnSpire, PerkinElmer, United States); then, the samples were diluted in tris-buffer 100 mM (pH 9) (Merck KGaA, Germany) to a final protein concentration of 0.5 mg/mL, and acetylcoenzyme A 100 μM and 5,5′-dithiobis-(2-nitrobenzoic) acid (DTNB) 100 μM were added. Oxaloacetic acid 500 μM was added to trigger the reaction. A microplate reader (EnSpire; PerkinElmer, United States) was used to measure the absorbance at 412 nm every 60 s for 30 min sets at 37°C. Citrate synthase activity was determined by interpolating the results using a calibration curve obtained by incubating known concentrations of the isolated enzyme (Merck KGaA, Germany) and using GraphPad Prism 8 for the calculations. Citrate synthase activity was expressed in mU/mL.

*Mitochondrial UCP-1:* Mitochondrial UCP-1 levels were measured following the instructions of Mouse UCP-1 ELISA Kit (E-EL-M0717-96T, CliniSciences, Italy) on cell supernatant.

### 2.4 Data analysis

The normal distribution of the dataset was tested using the Shapiro–Wilk test. Data are expressed as mean ± standard error (SEM). One-way ANOVA followed by Tukey’s *post hoc* test was performed as statistical analysis to compare the different lipid accumulations at each time point comparing the effect of insulin, RGZ 0.1 μM, RGZ 1 μM, ISO 0.1 μM, and ISO 1 μM versus preadipocytes (T0) (Figure 3B). One-way ANOVA followed by Tukey’s *post hoc* test was performed as statistical analysis to compare the effect of RGZ and ISO at each time point versus the white adipocyte phenotype (Figures 3–5; Figure 6A; Figure 7A). Two-way ANOVA followed by Tukey’s *post hoc* test was performed to analyze the effect of RGZ and ISO considering the differentiation timing by comparing the whole trends with the white adipocyte phenotype curve (Figures 6B, 7B). Statistically significant differences were defined as p < 0.05 (software: GraphPad Prism 8.0.2).

## 3 Results

### 3.1 Morphological and lipid deposit assessments

**FIGURE 2:**
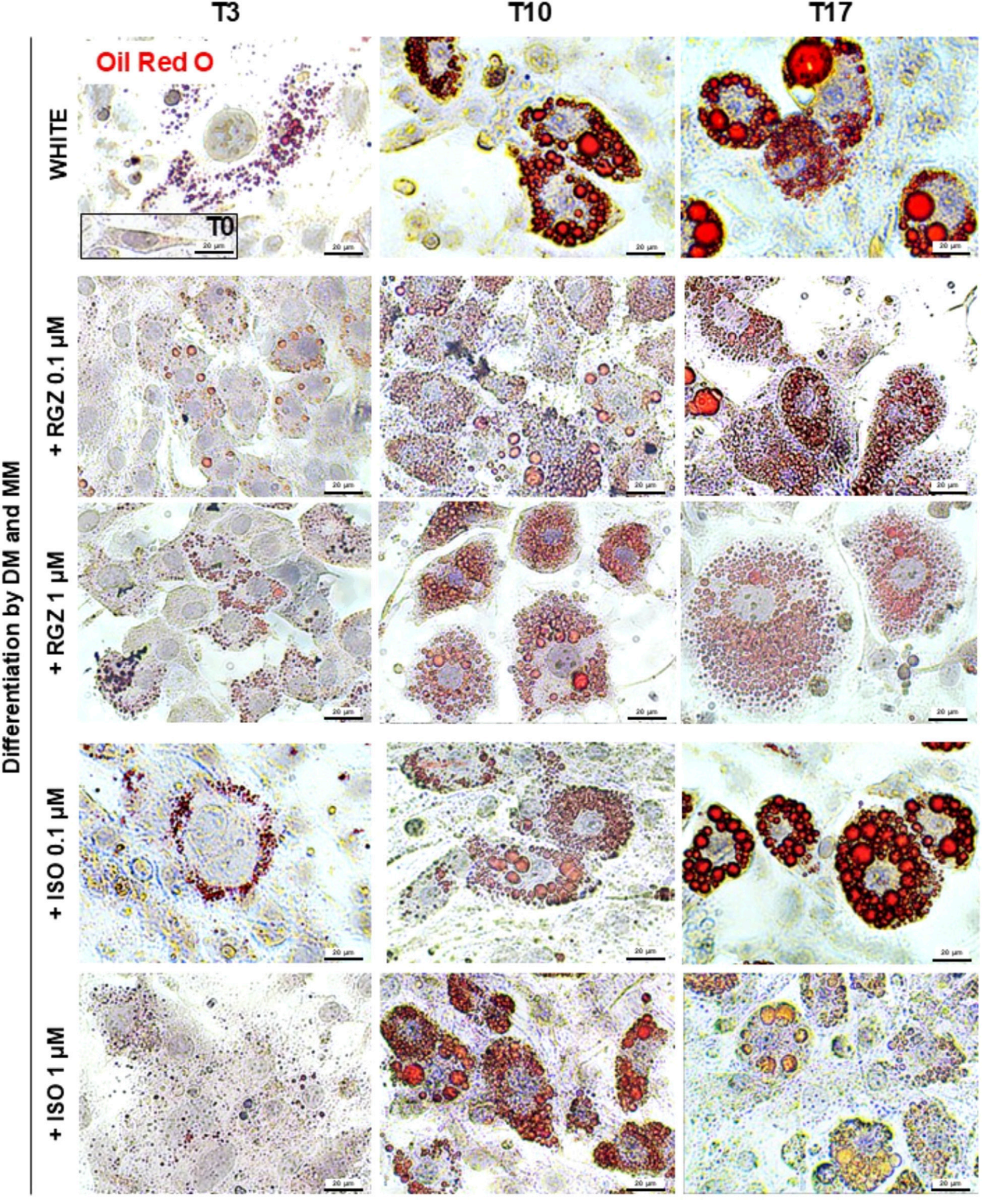
Representative images of ORO staining in preadipocytes (T0) and adipocytes differentiated at T3, T10, and T17 following treatment with DM and MM without or with RGZ 0.1 µM and 1 μM and ISO 0.1 µM and 1 µM. For each group and each time point, the cells contained in 10 fields acquired at ×40 magnification for image analysis of Oil Red O expression have been analyzed. Scale bar: 20 µm. Abbreviations: DM, differentiation medium; MM, maintenance medium; ISO, isoproterenol; RGZ, rosiglitazone; ORO, Oil Red O.

**FIGURE 3:**
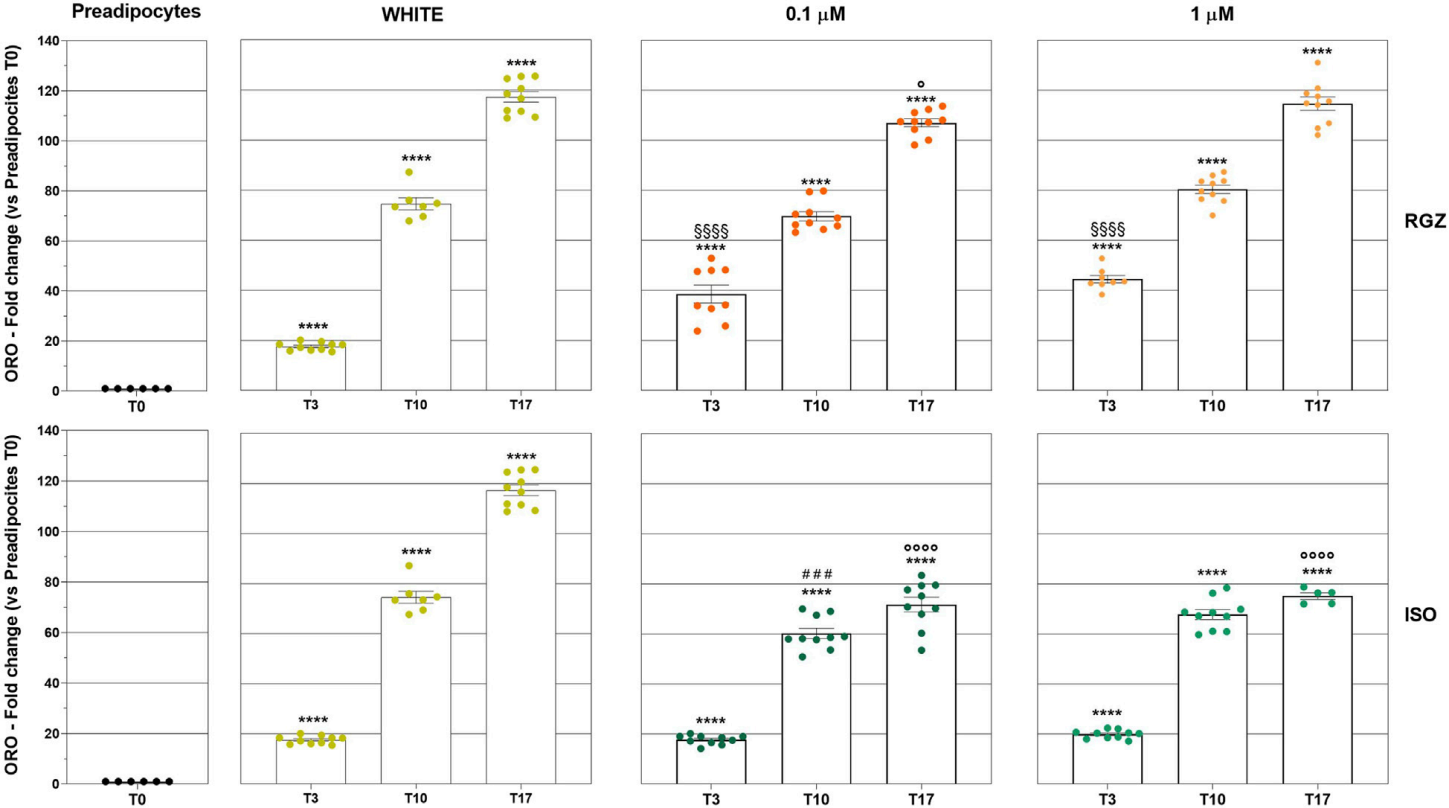
Quantitative analysis of lipidic deposits. Column graphs display the fold changes of the ORO staining expressed in percentage of positive pixels (PPP) ± SEM: statistical significance of adipocytes differentiated at T3, T10, and T17 following treatment with DM and MM (WHITE), RGZ 0.1 µM and 1 µM, or ISO 0.1 µM and ISO 1 µM versus T0 (****p < 0.0001). The graph also reports the statistical analysis of RGZ 0.1 μM T3 and RGZ 1 μM T3 versus WHITE T3 (§§§§p < 0.0001); ISO 0.1 μM T10 versus WHITE T10 (###p < 0.001); RGZ 0.1 µM T17, ISO 0.1 μM T17, and ISO 1 μM T17 versus WHITE T17 (°p < 0.05; °p < 0.0001). Abbreviations: ISO, isoproterenol; RGZ, rosiglitazone; ORO, Oil Red O.

Murine preadipocyte cells, derived from 3T3-L1 fibroblasts treated with DM, progressively modified their phenotype (Figure 2). At T0, the cells showed an elongated morphology with a negligible amount of ORO-positive intracellular lipid deposits, and they progressively started to trigger the lipid accumulation process, producing small lipid droplets (T3), which increased in size (T10) reaching even more significant ORO-positive drops at T17 (Figures 2, 3). Differentiation towards a white phenotype is also morphologically evident with the displacement of the nucleus from the center to the periphery of the cells. 3T3-L1 cells treated with RGZ 0.1 and 1 µM showed a significant increase in ORO-positive droplets starting from T3 (Figures 2, 3) to T17, with a progressive increase in the number of small lipid droplets surrounding the nucleus in an eccentric position, indicative of the maturation process towards the beige phenotype. ISO 0.1 µM and 1 µM induced a significant increase in ORO-positive cells starting from T3 (Figures 2, 3) but a lower grade of transition to the beige phenotype at all times compared to RGZ.

**FIGURE 4:**
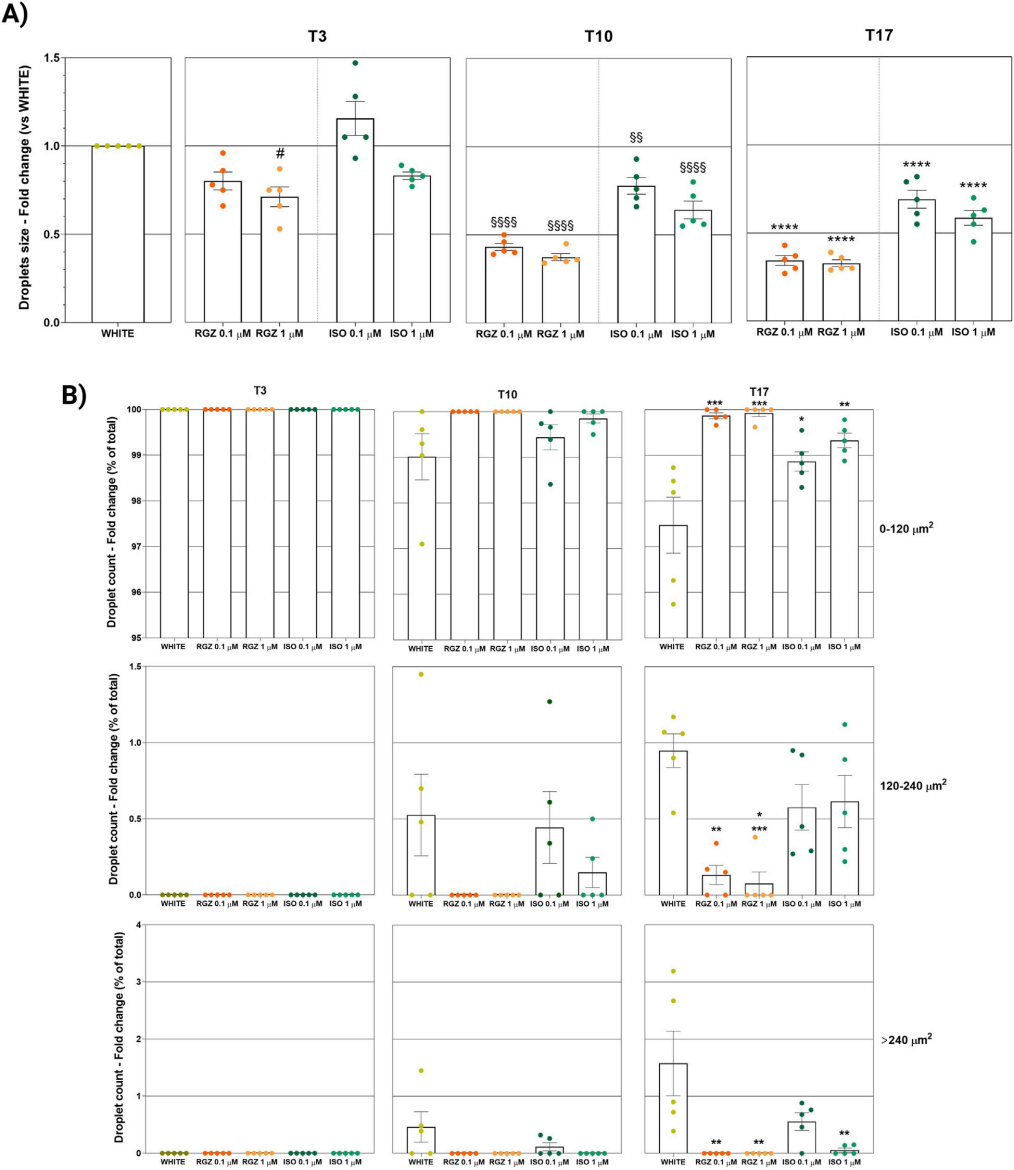
**(A)** Quantitative analysis of lipidic droplet size. Column graphs display the fold changes of the ORO-positive droplet size expressed as an average of all single areas ± SEM. Statistical significance of RGZ 0.1 μM T3 versus WHITE T3 (#p < 0.05); RGZ 0.1 µM T10, RGZ 1 μM T10, ISO 0.1 μM T10, and ISO 1 μM T10 versus WHITE T10 (§§p < 0.01; §§§§p < 0.0001); RGZ 0.1 µM T17, RGZ 1 μM T17, ISO 0.1 μM T17, and ISO 1 μM T17 versus WHITE T17 (****p < 0.0001). Abbreviations: ISO, isoproterenol; RGZ, rosiglitazone; ORO, Oil Red O. **(B)** Classification of lipidic droplet size. Column graphs display the fold changes of the ORO-positive droplet size expressed as a percentage of the ratio between the number of droplets with small, medium, and large size (0–120 μm^2^, 120 μm^2^–240 µm^2^, >240 μm^2^, respectively) and the total number of droplet counts for each group ± SEM. Five fields were evaluated for lipid droplet size in each group and at each time point. Statistical significance of RGZ 0.1 µM T17, RGZ 1 μM T17, ISO 0.1 μM T17 versus WHITE T17 (*p < 0.05; **p < 0.01; ***p < 0.001). Abbreviations: ISO, isoproterenol; RGZ, rosiglitazone; ORO, Oil Red O.

Quantitative analyses were also carried out to evaluate the size and lipid droplet size distribution in the different treatments. In particular, RGZ 0.1 μM and 1 μM treatments produced a cellular phenotype with lipid droplets that were significantly smaller than the white phenotype and that reduced their size in the progression of the time points (Figure 4A). In addition, the size of droplets was always significantly smaller than those present in cells treated with ISO 0.1 μM and 1 μM.

The success of the differentiation process with RGZ treatment was also highlighted by the analysis of the classification of the size of lipid droplets (Figure 4B). At T3, all droplets were small in all treatments. At T10, only RGZ kept the small droplets, while ISO showed a percentage of medium droplets comparable to those with the white phenotype and some large droplets (no significant changes were highlighted). At the last time point, T17, there was a small increase in the percentage of medium droplets with RGZ, a larger percentage of medium droplets with ISO, and maximum values in the white phenotype. The percentage of large droplets was significantly lower for RGZ 0.1 μM and 1 μM and ISO 1 μM at T17.

### 3.2 Evaluation of cellular and mitochondrial metabolic activity (citrate synthase)

**FIGURE 5:**
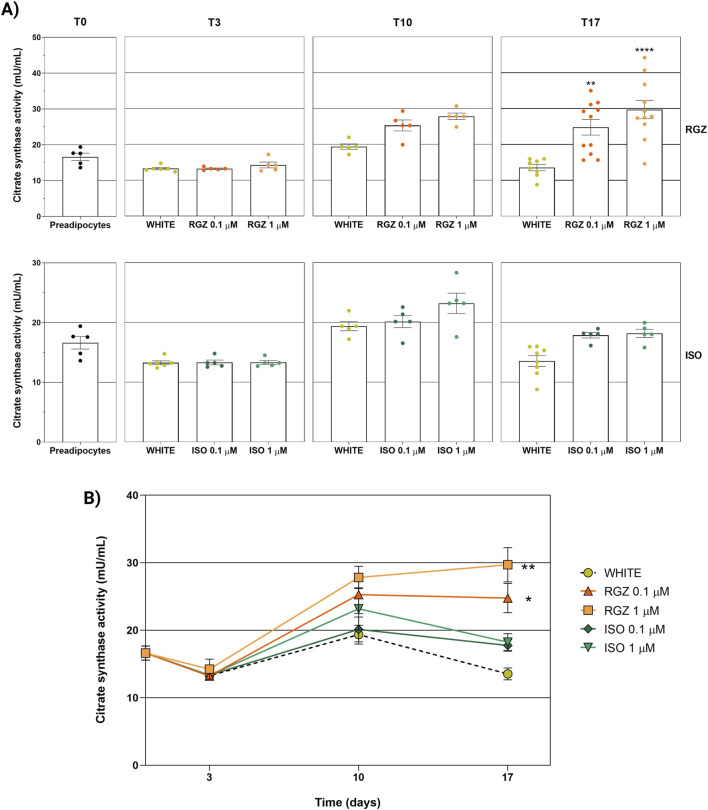
Analysis of UCP-1 levels used as a marker of the adipocyte phenotype at different differentiation times and following the different treatments used. **(A)** UCP-1 levels stratified by time (T0, T3, T10, and T17) and by treatment (RGZ 0.1 μM, RGZ 1 μM, ISO 0.1 μM, and ISO 1 μM). **(B)** Representation of the variation of UCP-1 levels as a function of time and stratified by treatment (RGZ 0.1 μM, RGZ 1 μM, ISO 0.1 μM, and ISO 1 μM). Graphs display the UCP-1 levels expressed in ng/mL ± SEM. Statistical significance versus WHITE T3 (#p < 0.05) in **(A)**. Statistical significance versus WHITE T10 (§§§p < 0.001; §§§§p < 0.0001) in **(A)**. Statistical significance versus WHITE T17 (****p < 0.0001) in **(A)**. Statistical significance versus WHITE (****p < 0.0001) in **(B)**. Abbreviations: ISO, isoproterenol; RGZ, rosiglitazone. Sample size (n): eight biological replicates for white T17 and four biological replicates for the other treatments. Three technical replicates per sample.

The white adipocyte phenotype showed low citrate synthase enzymatic activity during all three differentiation times evaluated (T3, T10, and T17). The results of white phenotype citrate synthase activity were comparable to undifferentiated cells, without statistically significant differences during the three differentiation times evaluated. ISO was unable to influence cellular metabolic activity at either tested concentration (0.1 μM and 1 μM) and at each time point evaluated. In contrast, RGZ promoted a statistically significant increase in mitochondrial enzymatic activity after 17 days of differentiation at the two concentrations of 0.1 μM and 1 μM (Figure 5A).

The analysis of the curves of the citrate synthase activity as a function of the differentiation time for each treatment and concentration used confirmed that RGZ promoted a statistically significant increase in enzymatic activity throughout the adipocyte differentiation process at both concentrations tested (0.1 μM and 1 μM). This analysis confirmed that ISO did not show any effects on the activity of the citrate synthase enzyme, highlighting a trend that overlaps the white adipocyte phenotype (Figure 5B).

### 3.3 Evaluation of UCP-1 levels and immunofluorescence localization

**FIGURE 6:**
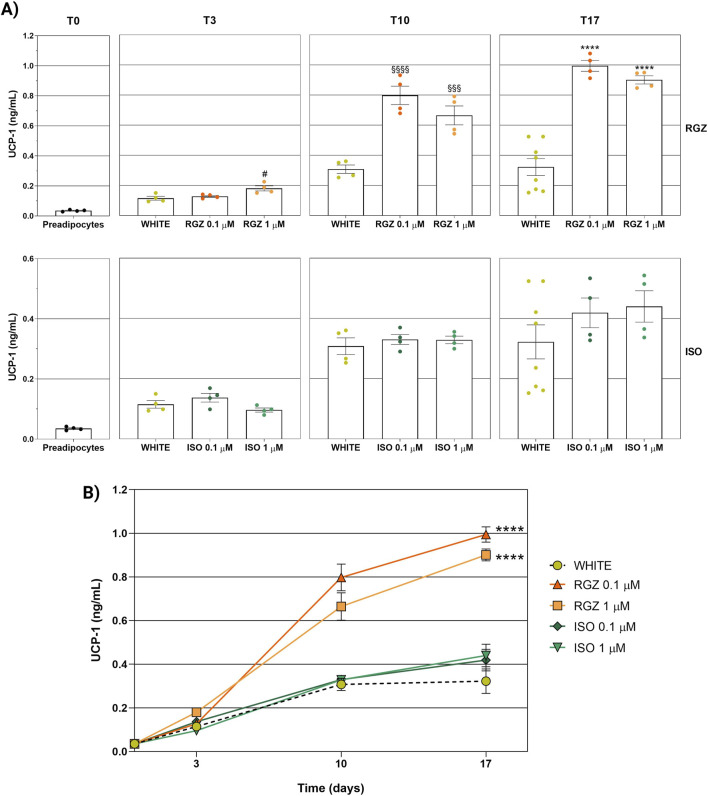
Analysis of the activity of the citrate synthase enzyme representing the metabolic and mitochondrial activity of adipocyte cells at different differentiation times and following the different treatments used. **(A)** Enzyme activity stratified by time (T0, T3, T10, and T17) and by treatment (RGZ 0.1 μM, RGZ 1 μM, ISO 0.1 μM, and ISO 1 μM). **(B)** Representation of the variation of enzymatic activity as a function of time and stratified by treatment (RGZ 0.1 μM, RGZ 1 μM, ISO 0.1 μM, and ISO 1 μM). Graphs display the citrate synthase activity expressed in mU/mL ± SEM. Statistical significance versus WHITE T17 (**p < 0.01; ****p < 0.0001) in **(A)**. Statistical significance versus WHITE (*p < 0.05; **p < 0.01) in **(B)**. Abbreviations: ISO, isoproterenol; RGZ, rosiglitazone. Sample size (n): six for white T3, five for white T10, eight biological replicates for white T17; five biological replicates for RGZ 0.1 μM, RGZ 1 μM, ISO 0.1 μM, and ISO 1 μM at T3 and T10; 11 biological replicates for RGZ 0.1 μM and RGZ 1 μM at T17, and five biological replicates for ISO 0.1 μM and ISO 1 μM at T17. Three technical replicates per sample.

UCP-1 levels were significantly increased in RGZ treatment at the highest concentration tested (RGZ 1 μM) starting from the differentiation step (T3) compared to the white phenotype. While the use of the maintenance cocktail used for the white phenotype led to a stabilization of UCP-1 levels after 10 and 17 days (T10 and T17), treatment with RGZ at both concentrations tested (0.1 μM and 1 μM) favored the increase of UCP-1 levels exceeding the values found in the white phenotype by 2.59-fold (T10-RGZ 0.1 μM), 2.16-fold (T10-RGZ 1 μM), 3.09-fold (T17-RGZ 0.1 μM), and 2.80-fold (T17-RGZ 1 μM) (Figures 6A, B). In this case, ISO did not show significant variations in UCP-1 levels for either concentration or differentiation timing (Figures 6A, B).

**FIGURE 7:**
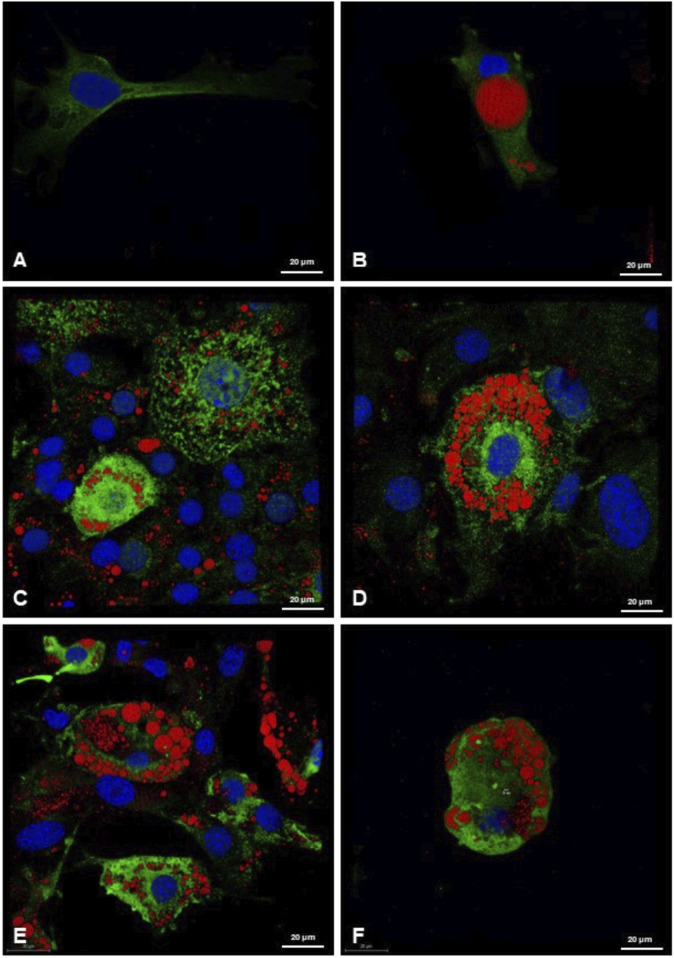
Representative 3D confocal images of lipid droplets (ORO, red) and UCP-1 (green) expression in **(A)** preadipocytes; **(B)** 3T3-L1 cells differentiated at T17 in white adipocytes, after treatment with DM and MM for white adipocyte differentiation; **(C, D)** following treatment with RGZ 0.1 µM and 1 μM; **(E)** and **(F)** following treatment with ISO 0.1 µM and 1 µM. Scale bar: 20 µM. Abbreviations: DM, differentiation medium; MM, maintenance medium; ISO, isoproterenol; RGZ, rosiglitazone; ORO, Oil Red O; UCP-1, mitochondrial uncoupling protein-1.

Immunofluorescence analysis showed a scarce presence of UCP-1-positive mitochondria in elongated preadipocytes and unilocular adipocytes with their single lipid droplets (Figures 7A, B). Differently, an abundance of UCP-1 protein expression was highlighted in 3T3-L1 differentiated cells, especially after the treatment with RGZ 0.1 μM and 1 μM at T17 in beige adipocytes, characterized by the multilocular morphology in which the lipid droplets thicken close together around the nucleus, which remains in an eccentric position (Figures 7C, D). Differentiation with ISO 0.1 μM and 1 μM at T17 in beige adipocytes has been shown to be less effective in morphological differentiation and in UCP-1 expression (Figures 7E, F). These data taken together confirm that the differentiation protocols into white and beige adipocytes have been very effective and that the most effective treatment for the transition to the beige phenotype is RGZ.

## 4 Discussion

Obesity and metabolic diseases are both consequences and predisposing factors for the morpho/functional and qualitative/quantitative degeneration of WAT (Hotamisligil, 2006; Meyer et al., 2013). Mitochondrial dysfunction, adipocyte hypertrophy, and increased reactive oxygen species (ROS) trigger hypoxia, increase metaflammation, and promote macrophage polarization (Crewe et al., 2017; Vishvanath and Gupta, 2019). These pathological conditions also negatively influence the “*de novo*” browning process that preadipocyte cells can undergo in response to physiological stimuli to ensure metabolic homeostasis and in response to exogenous stimulation. Exposure to low temperatures and physical exercise stimulates the browning process, as confirmed by several preclinical and clinical studies. In addition, also the exogenous activation of specific pathways, such as β-adrenergic and PPAR-γ activation, are reported, mainly by preclinical studies, to promote the browning process (Ziqubu et al., 2023). The availability of detailed and reproducible preclinical cellular experimental models is essential to analyze the activation of the “*de novo*” browning process of adipocyte precursors in WAT depots as an anti-obesity pharmacological strategy.

Based on these premises, 3T3-L1 cells, widely used in preclinical practice for adipocyte differentiation models and for the study of adipogenesis processes (Cave and Crowther, 2019), have been used to develop a model of adipocyte differentiation for investigating the “*de novo*” browning process. The 3T3-L1 cell line is already used for the screening of new molecules, or off-label properties of known molecules, to modulate the browning process. However, a defined protocol that temporally describes the obtained adipocyte phenotype (white or beige) and allows the reproducibility necessary for comparing the results obtained in different studies is still missing. Furthermore, the presence of a reference drug able to induce the *de novo* browning process leading to a beige adipocyte phenotype is necessary for validating the model, allowing the obtainment of more consistent results in investigating new potential molecules such as browning inducers. To this end, RGZ and ISO, activators of the most pharmacological stimuli described in inducing the browning process, the PPAR-γ and β-adrenergic pathways, respectively, have been investigated as possible reference drugs (Zhang et al., 2023; Machado et al., 2022).

The aim of this work was to add one more piece to a widely used adipocyte differentiation model of 3T3-L1 cells, characterizing the representative timepoints of the differentiation process itself. T10 was therefore selected to reproduce the timing mainly used by adipocyte differentiation protocols (Kaczmarek et al., 2024); T17 was analyzed to evaluate whether 10 days of differentiation were actually sufficient to obtain a mature beige adipocyte phenotype with the use of a single pharmacological stimulus such as RGZ or ISO to identify a suitable reference compound for the induction of the *de novo* differentiation process as well. During this time window, morphological and biochemical aspects were described at different representative time points (T0, T3, T10, T17) essential to define the adipocyte phenotype obtained.

T0 represents the preadipocyte stage after reaching a condition of over-confluence necessary for replicative arrest and the triggering of the differentiation process. This step revealed elongated cells and intracellular lipid deposits, confirming a preadipocyte phenotype.

T3 describes the changes triggered by the adipocyte differentiation cocktails reported in Table 2. In this step, morphological analysis did not highlight significant changes in cell morphology compared with T0 and the first, albeit small, signs of lipid recruitment begin to emerge. The enzymatic activity of citrate synthase does not show significant variations compared to the values found in preadipocyte cells for any analyzed treatments (insulin, RGZ 0.1 μM, RGZ 1 μM, ISO 0.1 μM, or ISO 1 μM). These data indicate that although there are no significant morphological variations, cellular differentiation has been started. Furthermore, the analysis of UCP-1 levels in the cell lysate highlights a positive trend in all the treatments. UCP-1 is responsible for the uncoupling of the mitochondrial respiration chain, and for this reason, it is particularly expressed in thermogenically active adipocytes such as BAT and BeAT. Its importance in the characterization of the experimental model for the induction of the browning process is therefore evident.

T10 could represent the turning point for the differentiation process. Indeed, the results obtained confirm that after 3 days of DM and 7 days of maintenance medium (MM), the cellular morphology is significantly modified, the intracellular lipid deposits are significantly increased compared to T3, assuming a multilocular conformation, and the cellular metabolic activity and the levels of UCP-1 are generally increased confirming the development of a cellular phenotype in the maturation phase. Although RGZ shows a reduction in the size of lipid deposits, no clear changes in the presence of medium/large lipid droplets and metabolic activity were observed with RGZ compared to the white adipocyte phenotype at the same time point T10, giving us a red flag on the actual achievement of a mature adipocyte phenotype. High levels of UCP-1 following RGZ treatment at both concentrations tested (0.1 and 1 μM) underline its positive profile in the modulation of the thermogenic pathway.

T17 represents the last analyzed checkpoint from which significant differences emerge compared to the beginning of the differentiation protocol (T3) and compared to T10. Cellular morphology is better defined and shows differences between the treatment groups. Insulin stimulation alone leads to an adipocyte phenotype characterized by high lipid deposits arranged in unilocular structures that occupy most of the body cell, with consequent displacement of the nucleus to the burden of the cell membrane. The cell shape is rounded, and the dimensions are increased. The enzymatic activity and UCP-1 underline the achievement of a mature white adipocyte phenotype characterized by low cellular and mitochondrial activity. The addition of ISO does not provide significant changes in cell morphology and lipid deposits, nor in the activity levels of citrate synthase and UCP-1, describing a phenotype overlapping with the white one. RGZ instead confirms the positive trend shown in the previous time point, leading to an adipocyte with reduced dimensions, multilocular arrangement of the lipid droplets, and a centrally located nucleus. Metabolic activity and UCP-1 levels are significantly increased compared to the phenotype obtained with insulin stimulation alone, confirming that a mature adipocyte phenotype overlapping with a thermogenically active beige adipocyte has been obtained.

It is important to underline that ISO, a non-selective β-agonist, has been selected to mimic a general stimulation of the sympathetic nervous system. The lack of efficacy of ISO found in these experimental conditions could, however, be due to an insufficient concentration or the lack of selectivity for β3 receptors that, as described by some experimental evidence, seems to be implicated in the browning process in WAT (Richard et al., 2017; Fan et al., 2019). In accordance with this hypothesis, a randomized controlled trial carried out on 32 subjects demonstrated the ability of Mirabegron, a selective β3 agonist on the market as a relaxant of the smooth muscles of the genitourinary tract, to promote the formation of BeAT in human sWAT, with results comparable to the subjects exposed to low temperatures, confirming the importance of β3 adrenergic stimulation (Ferrere et al., 2021; Finlin et al., 2020). From the results obtained, it is therefore clear that a non-selective β-adrenergic stimulation fails to promote the browning process, providing valuable insights for the investigation of a selective stimulation of the β3 isoform.

The results obtained from our experimental procedures are in line with a marked effect of RGZ and, in general, of PPAR-γ agonists in the positive modulation of the browning process of human adipocyte cells. Experimental evidence performed on human multipotent adipose-derived stem cells (hMADS) showed a significant increase in UCP-1 levels at day 16 of differentiation in the presence of RGZ 0.1 μM. UCP-1 levels analyzed after 10 days of differentiation in the presence of RGZ 0.1 μM showed a positive trend that did not reach statistical significance, highlighting a higher variability than the data obtained on the 16th day. This further overlaps with the data achieved in this work, confirming the need to further clarify some details to optimize the experimental protocols of adipocyte differentiation to study the browning process and, above all, to be able to test new pharmacological approaches for the modulation of the *de novo* browning process (Pisani et al., 2011).

Overall, the results confirm the importance of RGZ in modulating the *de novo* browning process. Qualitative/quantitative results are in line with those highlighted in adipocyte differentiation models on human-immortalized preadipocyte (Chub-S7), primary human pre-adipocytes, and human multipotent adipose-derived stem cells (hMADS) where an increase in UCP-1 levels and mitochondrial metabolic activity, as well as a clear definition of a beige adipocyte phenotype, were made possible by the addition of RGZ (Pisani et al., 2011; Harms et al., 2019; Leyvraz et al., 2010).

In conclusion, the results obtained in this work led to a detailed description of the morphological changes of preadipocyte cells during the 17-day differentiation protocol used, as well as the characterization of the mature adipocyte phenotypes obtained in these experimental conditions. What emerges from the data leads to identifying RGZ as an excellent modulator of the browning process and as an ideal reference drug for the induction of the *de novo* browning process in the experimental conditions used. The use of RGZ for 10 days seems capable of significantly increasing some of the key parameters to identify a mature beige adipocyte phenotype, even if some results obtained do not show statistical significance (citrate synthase activity) or a clear scenario useful to discriminate through different phenotypes. These results suggest that at day 10 of RGZ differentiation, we could see a significant modification of lipid accumulation but miss a mature adipocyte phenotype (either white or beige). On the 10th day, we might still be in an intermediate stage where the preadipocyte population has not reached a complete differentiation step. The use of RGZ for 17 days, instead, seems more suitable for the study of the *de novo* browning process and the possible exogenous modulatory strategy, highlighting better data accuracy and a clear statistical significance for each parameter evaluated.

## Data Availability

The original contributions presented in the study are included in the article/Supplementary Material; further inquiries can be directed at the corresponding author.

## References

[B1] Asano H. Kanamori Y. Higurashi S. Nara T. Kato K. Matsui T. (2014). Induction of beige-like adipocytes in 3T3-L1 cells. J. Veterinary Med. Sci. 76 (1), 57–64. 10.1292/jvms.13-0359 PMC3979956 24065084

[B2] Bartelt A. Heeren J. (2014). Adipose tissue browning and metabolic health. Nat. Rev. Endocrinol. 10 (1), 24–36. 10.1038/nrendo.2013.204 24146030

[B3] Brownstein A. J. Veliova M. Acin-Perez R. Liesa M. Shirihai O. S. (2022). ATP-consuming futile cycles as energy dissipating mechanisms to counteract obesity. Rev. Endocr. Metabolic Disord. 23 (1), 121–131. 10.1007/s11154-021-09690-w PMC8873062 34741717

[B4] Cannon B. Nedergaard J. (2004). Brown adipose tissue: function and physiological significance. Physiol. Rev. 84, 277–359. 10.1152/physrev.00015.2003 14715917

[B5] Cave E. Crowther N. J. (2019). “The use of 3T3-L1 murine preadipocytes as a model of adipogenesis,” in Pre-clinical models: techniques and protocols, 263–272. 10.1007/978-1-4939-8994-2_25 30535703

[B6] Chen J. Yang Y. Li S. Yang Y. Dai Z. Wang F. (2020). E2F1 regulates adipocyte differentiation and adipogenesis by activating ICAT. Cells 9 (4), 1024. 10.3390/cells9041024 32326181 PMC7225968

[B7] Choi M. Mukherjee S. Yun J. W. (2021). Trigonelline induces browning in 3T3‐L1 white adipocytes. Phytotherapy Res. 35 (2), 1113–1124. 10.1002/ptr.6892 33015893

[B8] Chouchani E. T. Kajimura S. (2019). Metabolic adaptation and maladaptation in adipose tissue. Nat. Metab. 1 (2), 189–200. 10.1038/s42255-018-0021-8 31903450 PMC6941795

[B9] Crewe C. An Y. A. Scherer P. E. (2017). The ominous triad of adipose tissue dysfunction: inflammation, fibrosis, and impaired angiogenesis. J. Clin. investigation 127 (1), 74–82. 10.1172/JCI88883 PMC5199684 28045400

[B10] Després J.-P. Lemieux I. (2006). Abdominal obesity and metabolic syndrome. Nature 444 (7121), 881–887. 10.1038/nature05488 17167477

[B11] Fan L. Xu H. Yang R. Zang Y. Chen J. Qin H. (2019). Combination of capsaicin and capsiate induces browning in 3T3-L1 white adipocytes via activation of the peroxisome proliferator-activated receptor γ/β3-adrenergic receptor signaling pathways. J. Agric. food Chem. 67 (22), 6232–6240. 10.1021/acs.jafc.9b02191 31075194

[B12] Ferrannini E. Iozzo P. Virtanen K. A. Honka M.-J. Bucci M. Nuutila P. (2018). Adipose tissue and skeletal muscle insulin-mediated glucose uptake in insulin resistance: role of blood flow and diabetes. Am. J. Clin. Nutr. 108 (4), 749–758. 10.1093/ajcn/nqy162 30239554

[B13] Ferrere G. Alou M. T. Liu P. Goubet A.-G. Fidelle M. Kepp O. (2021). Ketogenic diet and ketone bodies enhance the anticancer effects of PD-1 blockade. JCI insight 6 (2), e145207. 10.1172/jci.insight.145207 33320838 PMC7934884

[B14] Finlin B. S. Memetimin H. Zhu B. Confides A. L. Vekaria H. J. El Khouli R. H. (2020). The β3-adrenergic receptor agonist mirabegron improves glucose homeostasis in obese humans. J. Clin. investigation 130 (5), 2319–2331. 10.1172/JCI134892 PMC7190997 31961829

[B15] Flori L. Piragine E. Spezzini J. Citi V. Calderone V. Martelli A. (2023). Influence of polyphenols on adipose tissue: sirtuins as pivotal players in the Browning process. Int. J. Mol. Sci. 24 (11), 9276. 10.3390/ijms24119276 37298226 PMC10253356

[B16] Ghigliotti G. Barisione C. Garibaldi S. Fabbi P. Brunelli C. Spallarossa P. (2014). Adipose tissue immune response: novel triggers and consequences for chronic inflammatory conditions. Inflammation 37, 1337–1353. 10.1007/s10753-014-9914-1 24823865 PMC4077305

[B17] Golozoubova V. Cannon B. Nedergaard J. (2006). UCP1 is essential for adaptive adrenergic nonshivering thermogenesis. Am. J. Physiology-Endocrinology Metabolism 291 (2), E350–E357. 10.1152/ajpendo.00387.2005 16595854

[B18] Granneman J. G. Li P. Zhu Z. Lu Y. (2005). Metabolic and cellular plasticity in white adipose tissue I: effects of beta3-adrenergic receptor activation. Am. J. Physiology-Endocrinology Metabolism 289 (4), E608–E616. 10.1152/ajpendo.00009.2005 15941787

[B19] Harms M. J. Li Q. Lee S. Zhang C. Kull B. Hallen S. (2019). Mature human white adipocytes cultured under membranes maintain identity, function, and can transdifferentiate into brown-like adipocytes. Cell Rep. 27 (1), 213–225. e5. 10.1016/j.celrep.2019.03.026 30943403

[B20] Hilton C. Karpe F. Pinnick K. E. (2015). Role of developmental transcription factors in white, brown and beige adipose tissues. Biochimica Biophysica Acta (BBA)-Molecular Cell Biol. Lipids 1851 (5), 686–696. 10.1016/j.bbalip.2015.02.003 25668679

[B21] Himms-Hagen J. Melnyk A. Zingaretti M. Ceresi E. Barbatelli G. Cinti S. (2000). Multilocular fat cells in WAT of CL-316243-treated rats derive directly from white adipocytes. Am. J. Physiology-Cell Physiology 279 (3), C670–C681. 10.1152/ajpcell.2000.279.3.C670 10942717

[B22] Hotamisligil G. S. (2006). Inflammation and metabolic disorders. Nature 444 (7121), 860–867. 10.1038/nature05485 17167474

[B23] Hotamisligil G. S. (2017). Inflammation, metaflammation and immunometabolic disorders. Nature 542 (7640), 177–185. 10.1038/nature21363 28179656

[B24] Huang L. Guo Z. Huang M. Zeng X. Huang H. (2024). Triiodothyronine (T3) promotes browning of white adipose through inhibition of the PI3K/AKT signalling pathway. Sci. Rep. 14 (1), 20370. 10.1038/s41598-024-71591-0 39223267 PMC11369215

[B25] Ikeda K. Yamada T. (2020). UCP1 dependent and independent thermogenesis in brown and beige adipocytes. Front. Endocrinol. 11, 498. 10.3389/fendo.2020.00498 PMC7399049 32849287

[B26] Kaczmarek I. Suchý T. Strnadová M. Thor D. (2024). Qualitative and quantitative analysis of lipid droplets in mature 3T3-L1 adipocytes using oil red O. Star. Protoc. 5 (2), 102977. 10.1016/j.xpro.2024.102977 38875117 PMC11225905

[B27] Kawai T. Autieri M. V. Scalia R. (2021). Adipose tissue inflammation and metabolic dysfunction in obesity. Am. J. Physiology-Cell Physiology 320 (3), C375–C391. 10.1152/ajpcell.00379.2020 PMC8294624 33356944

[B28] Keipert S. Kutschke M. Lamp D. Brachthäuser L. Neff F. Meyer C. W. (2015). Genetic disruption of uncoupling protein 1 in mice renders brown adipose tissue a significant source of FGF21 secretion. Mol. Metab. 4 (7), 537–542. 10.1016/j.molmet.2015.04.006 26137441 PMC4481421

[B29] Kershaw E. E. Flier J. S. (2004). Adipose tissue as an endocrine organ. J. Clin. Endocrinol. and Metabolism 89 (6), 2548–2556. 10.1210/jc.2004-0395 15181022

[B30] Kim K. Nam K. H. Yi S. A. Park J. W. Han J.-W. Lee J. (2020). Ginsenoside Rg3 induces browning of 3T3-L1 adipocytes by activating AMPK signaling. Nutrients 12 (2), 427. 10.3390/nu12020427 32046061 PMC7071202

[B31] Lee H. S. Choi S. M. Lim S. H. Choi C.-I. (2023b). Betanin from beetroot (beta vulgaris L.) regulates lipid metabolism and promotes fat browning in 3T3-L1 adipocytes. Pharmaceuticals 16 (12), 1727. 10.3390/ph16121727 38139853 PMC10748323

[B32] Lee H. S. Heo C. U. Song Y.-H. Lee K. Choi C.-I. (2023a). Naringin promotes fat browning mediated by UCP1 activation via the AMPK signaling pathway in 3T3-L1 adipocytes. Archives Pharmacal Res. 46 (3), 192–205. 10.1007/s12272-023-01432-7 36840853

[B33] Leyvraz C. Suter M. Verdumo C. Calmes J. M. Paroz A. Darimont C. (2010). Selective effects of PPARgamma agonists and antagonists on human pre-adipocyte differentiation. Diabetes, Obes. Metabolism 12 (3), 195–203. 10.1111/j.1463-1326.2009.01149.x 19895635

[B34] Lim S. Honek J. Xue Y. Seki T. Cao Z. Andersson P. (2012). Cold-induced activation of brown adipose tissue and adipose angiogenesis in mice. Nat. Protoc. 7 (3), 606–615. 10.1038/nprot.2012.013 22383039

[B35] Lowell B. B. S-Susulic V. Hamann A. Lawitts J. A. Himms-Hagen J. Boyer B. B. (1993). Development of obesity in transgenic mice after genetic ablation of brown adipose tissue. Nature 366 (6457), 740–742. 10.1038/366740a0 8264795

[B36] Machado S. A. Pasquarelli-do-Nascimento G. Da Silva D. S. Farias G. R. de Oliveira Santos I. Baptista L. B. (2022). Browning of the white adipose tissue regulation: new insights into nutritional and metabolic relevance in health and diseases. Nutr. and metabolism 19 (1), 61. 10.1186/s12986-022-00694-0 PMC9446768 36068578

[B37] Madan K. Paliwal S. Sharma S. Kesar S. Chauhan N. Madan M. (2023). Metabolic syndrome: the constellation of co-morbidities, a global threat. Endocr. Metabolic and Immune Disorders-Drug Targets Formerly Curr. Drug Targets-Immune, Endocr. and Metabolic Disord. 23 (12), 1491–1504. 10.2174/1871530323666230309144825 36892127

[B38] Meyer L. K. Ciaraldi T. P. Henry R. R. Wittgrove A. C. Phillips S. A. (2013). Adipose tissue depot and cell size dependency of adiponectin synthesis and secretion in human obesity. Adipocyte 2 (4), 217–226. 10.4161/adip.24953 24052897 PMC3774697

[B39] Miller C. N. Yang J.-Y. England E. Yin A. Baile C. A. Rayalam S. (2015). Isoproterenol increases uncoupling, glycolysis, and markers of beiging in mature 3T3-L1 adipocytes. PloS one 10 (9), e0138344. 10.1371/journal.pone.0138344 26390217 PMC4577088

[B40] Mohamed-Ali V. Pinkney J. Coppack S. (1998). Adipose tissue as an endocrine and paracrine organ. Int. J. Obes. 22 (12), 1145–1158. 10.1038/sj.ijo.0800770 9877249

[B41] Molinari F. Feraco A. Mirabilii S. Saladini S. Sansone L. Vernucci E. (2021). SIRT5 inhibition induces brown fat-like phenotype in 3T3-L1 preadipocytes. Cells 10 (5), 1126. 10.3390/cells10051126 34066961 PMC8148511

[B42] Monteiro R. Azevedo I. (2010). Chronic inflammation in obesity and the metabolic syndrome. Mediat. Inflamm. 2010 (1), 289645. 10.1155/2010/289645 PMC2913796 20706689

[B43] Mu W.-J. Zhu J.-Y. Chen M. Guo L. (2021). Exercise-mediated browning of white adipose tissue: its significance, mechanism and effectiveness. Int. J. Mol. Sci. 22 (21), 11512. 10.3390/ijms222111512 34768943 PMC8583930

[B44] Nøhr M. K. Bobba N. Richelsen B. Lund S. Pedersen S. B. (2017). Inflammation downregulates UCP1 expression in brown adipocytes potentially via SIRT1 and DBC1 interaction. Int. J. Mol. Sci. 18 (5), 1006. 10.3390/ijms18051006 28481291 PMC5454919

[B45] Pacifici F. Farias C. L. A. Rea S. Capuani B. Feraco A. Coppola A. (2020). Tyrosol may prevent obesity by inhibiting adipogenesis in 3T3‐L1 preadipocytes. Oxidative Med. Cell. Longev. 2020 (1), 4794780. 10.1155/2020/4794780 PMC7746459 33376578

[B46] Pilkington A.-C. Paz H. A. Wankhade U. D. (2021). Beige adipose tissue identification and marker Specificity—overview. Front. Endocrinol. 12, 599134. 10.3389/fendo.2021.599134 PMC7996049 33776911

[B47] Pisani D. F. Djedaini M. Beranger G. E. Elabd C. Scheideler M. Ailhaud G. (2011). Differentiation of human adipose-derived stem cells into “brite”(brown-in-white) adipocytes. Front. Endocrinol. 2, 87. 10.3389/fendo.2011.00087 PMC3356055 22654831

[B48] Prins J. B. (2002). Adipose tissue as an endocrine organ. Best Pract. and Res. Clin. Endocrinol. and Metabolism 16 (4), 639–651. 10.1053/beem.2002.0222 12468412

[B49] Puri V. Ranjit S. Konda S. Nicoloro S. M. Straubhaar J. Chawla A. (2008). Cidea is associated with lipid droplets and insulin sensitivity in humans. Proc. Natl. Acad. Sci. 105 (22), 7833–7838. 10.1073/pnas.0802063105 18509062 PMC2409392

[B50] Recinella L. De Filippis B. Libero M. L. Ammazzalorso A. Chiavaroli A. Orlando G. (2023). Anti-inflammatory, antioxidant, and WAT/BAT-Conversion stimulation induced by novel PPAR ligands: results from *ex vivo* and *in vitro* studies. Pharmaceuticals 16 (3), 346. 10.3390/ph16030346 36986448 PMC10056895

[B51] Richard J. E. López-Ferreras L. Chanclón B. Eerola K. Micallef P. Skibicka K. P. (2017). CNS β3-adrenergic receptor activation regulates feeding behavior, white fat browning, and body weight. Am. J. Physiology-Endocrinology Metabolism 313 (3), E344–E358. 10.1152/ajpendo.00418.2016 28588096

[B52] Roberto C. A. Swinburn B. Hawkes C. Huang T. T. Costa S. A. Ashe M. (2015). Patchy progress on obesity prevention: emerging examples, entrenched barriers, and new thinking. Lancet 385 (9985), 2400–2409. 10.1016/S0140-6736(14)61744-X 25703111

[B53] Roberts‐Toler C. O'Neill B. T. Cypess A. M. (2015). Diet‐induced obesity causes insulin resistance in mouse brown adipose tissue. Obesity 23 (9), 1765–1770. 10.1002/oby.21134 26242777 PMC4551605

[B54] Sabaratnam R. Hansen D. R. Svenningsen P. (2023). White adipose tissue mitochondrial bioenergetics in metabolic diseases. Rev. Endocr. Metabolic Disord. 24 (6), 1121–1133. 10.1007/s11154-023-09827-z 37558853

[B55] Sakamoto T. Nitta T. Maruno K. Yeh Y.-S. Kuwata H. Tomita K. (2016). Macrophage infiltration into obese adipose tissues suppresses the induction of UCP1 level in mice. Am. J. Physiology-Endocrinology Metabolism 310 (8), E676–E687. 10.1152/ajpendo.00028.2015 26884382

[B56] Scheja L. Heeren J. (2019). The endocrine function of adipose tissues in health and cardiometabolic disease. Nat. Rev. Endocrinol. 15 (9), 507–524. 10.1038/s41574-019-0230-6 31296970

[B57] Seale P. Kajimura S. Spiegelman B. M. (2009). Transcriptional control of brown adipocyte development and physiological function—of mice and men. Genes and Dev. 23 (7), 788–797. 10.1101/gad.1779209 19339685 PMC2763499

[B58] Seo Y.-J. Kim K.-J. Choi J. Koh E.-J. Lee B.-Y. (2018). Spirulina maxima extract reduces obesity through suppression of adipogenesis and activation of browning in 3T3-L1 cells and high-fat diet-induced obese mice. Nutrients 10 (6), 712. 10.3390/nu10060712 29865208 PMC6024816

[B59] Silva J. E. (2011). Physiological importance and control of non-shivering facultative thermogenesis. Front. Biosci. Sch. Ed. 3 (1), 352–371. 10.2741/s156 21196381

[B60] Šimják P. Anderlová K. Cinkajzlová A. Pařízek A. Kršek M. Haluzík M. (2020). The possible role of endocrine dysfunction of adipose tissue in gestational diabetes mellitus. Minerva Endocrinol. 45 (3), 228–242. 10.23736/S0391-1977.20.03192-2 33000620

[B61] Stanford K. I. Middelbeek R. J. Townsend K. L. An D. Nygaard E. B. Hitchcox K. M. (2012). Brown adipose tissue regulates glucose homeostasis and insulin sensitivity. J. Clin. investigation 123 (1), 215–223. 10.1172/JCI62308 PMC3533266 23221344

[B62] Stolarczyk E. (2017). Adipose tissue inflammation in obesity: a metabolic or immune response? Curr. Opin. Pharmacol. 37, 35–40. 10.1016/j.coph.2017.08.006 28843953

[B63] Takanezawa Y. Nakamura R. Ohshiro Y. Uraguchi S. Kiyono M. (2023). Gadolinium-based contrast agents suppress adipocyte differentiation in 3T3-L1 cells. Toxicol. Lett. 383, 196–203. 10.1016/j.toxlet.2023.07.003 37437671

[B64] Ukropec J. Anunciado R. P. Ravussin Y. Hulver M. W. Kozak L. P. (2006). UCP1-independent thermogenesis in white adipose tissue of cold-acclimated Ucp1-/-mice. J. Biol. Chem. 281 (42), 31894–31908. 10.1074/jbc.M606114200 16914547

[B65] Unamuno X. Gómez‐Ambrosi J. Rodríguez A. Becerril S. Frühbeck G. Catalán V. (2018). Adipokine dysregulation and adipose tissue inflammation in human obesity. Eur. J. Clin. investigation 48 (9), e12997. 10.1111/eci.12997 29995306

[B66] Villarroya F. Cereijo R. Gavaldà‐Navarro A. Villarroya J. Giralt M. (2018). Inflammation of brown/beige adipose tissues in obesity and metabolic disease. J. Intern. Med. 284 (5), 492–504. 10.1111/joim.12803 29923291

[B67] Vishvanath L. Gupta R. K. (2019). Contribution of adipogenesis to healthy adipose tissue expansion in obesity. J. Clin. investigation 129 (10), 4022–4031. 10.1172/JCI129191 PMC6763245 31573549

[B68] Xu S. Lu F. Gao J. Yuan Y. (2024). Inflammation‐mediated metabolic regulation in adipose tissue. Obes. Rev. 25 (6), e13724. 10.1111/obr.13724 38408757

[B69] Yang M Liu S Zhang C. (2022). The related metabolic diseases and treatments of obesity. Healthcare (Basel). 10 (9), 1616. 10.3390/healthcare10091616 36141228 PMC9498506

[B70] Zebisch K. Voigt V. Wabitsch M. Brandsch M. (2012). Protocol for effective differentiation of 3T3-L1 cells to adipocytes. Anal. Biochem. 425 (1), 88–90. 10.1016/j.ab.2012.03.005 22425542

[B71] Zhang P. Su L. Ji X. Ma F. Yue Q. Zhao C. (2022). Cistanche promotes the adipogenesis of 3T3-L1 preadipocytes. Plos one 17 (3), e0264772. 10.1371/journal.pone.0264772 35231074 PMC8887766

[B72] Zhang Q. He C.-X. Wang L.-Y. Qian D. Tang D.-D. Jiang S.-N. (2023). Hydroxy-α-sanshool from the fruits of Zanthoxylum bungeanum Maxim. promotes browning of white fat by activating TRPV1 to induce PPAR-γ deacetylation. Phytomedicine Int. J. phytotherapy Phytopharm. 121, 155113. 10.1016/j.phymed.2023.155113 37748388

[B73] Ziqubu K. Dludla P. V. Mthembu S. X. Nkambule B. B. Mabhida S. E. Jack B. U. (2023). An insight into brown/beige adipose tissue whitening, a metabolic complication of obesity with the multifactorial origin. Front. Endocrinol. 14, 1114767. 10.3389/fendo.2023.1114767 PMC9978510 36875450

[B74] Zwick R. K. Guerrero-Juarez C. F. Horsley V. Plikus M. V. (2018). Anatomical, physiological, and functional diversity of adipose tissue. Cell metab. 27 (1), 68–83. 10.1016/j.cmet.2017.12.002 29320711 PMC6050204

